# The quality of life when a partner has substance use problems: a scoping review

**DOI:** 10.1186/s12955-018-1042-4

**Published:** 2018-11-20

**Authors:** Bente Birkeland, Kim Foster, Anne S. Selbekk, Magnhild M. Høie, Torleif Ruud, Bente Weimand

**Affiliations:** 1 Addiction Department, Research Unit, Sørlandet Hospital HF, P.B. 416, 4604 Kristiansand, Norway; 2 School of Nursing, Midwifery & Paramedicine, Australian Catholic University, Victoria, Australia; 3 NorthWestern Mental Health, Victoria, Australia; 4 Department for Research and Development, Rogaland A-senter, Stavanger, Norway; 5 Department of Psychosocial Health, Faculthy of Health and Sports Science, University of Agder, Grimstad, Norway; 6 Institute of Clinical Medicine, University of Oslo, Oslo, Norway; 7 Division Mental Health Services, R&D Department, Akershus University Hospital, 1478, Lørenskog, Norway; 8 Faculty of Health Sciences, Oslo Metropolitan University, Oslo, Norway

**Keywords:** Quality of life, Partners, Substance use

## Abstract

**Objective:**

To examine the existing body of knowledge on quality of life (QoL) in partners of people with substance use problems (PP-SUPs) to provide a synthesized summary of the evidence and identify gaps in our knowledge on the QoL of PP-SUPs**.**

**Methods:**

A systematic scoping review was performed. Publications indexed in EMBASE, Medline, PsycINFO, CINAHL, SocINDEX, and CENTRAL were searched for original, empirical, peer-reviewed, full-length research papers that examined QoL in PP-SUPs. Research papers identified through a manual search of key references and known references by co-authors were also included. A total of 3070 abstracts were screened, 41 full-text papers examined, and nine were found to meet the inclusion criteria. Eligibility was determined in two steps by four and two independent researchers, respectively. The main findings were explored by content analysis.

**Results:**

Eight of the nine included studies had quantitative designs, one had a mixed methods design, and no qualitative studies were found. Three studies were conducted exclusively among PP-SUPs, whereas the others included various subgroups. A majority of participants were women, and no study was conducted exclusively among men. Nearly half of the studies reported on whether there were minor children in the PP-SUPs’ household. The studies used established and generic QoL instruments based on different conceptual and theoretical perspectives on QoL. A majority of the studies found lower QoL in PP-SUPs than in general population, with substance use by the person with a SUP having the most impact on QoL of all evaluated factors. Two studies reported that gender was associated with QoL, with poor QoL being associated with being a male partner and vice versa for female partners.

**Conclusions:**

Further research is needed to examine QoL in PP-SUPs exclusively. A variety of QoL instruments covering various, but limited, dimensions of the concept have been used in previous studies of PP-SUPs. Thus, obtaining a comprehensive understanding of PP-SUPs’ QoL is challenging. Both qualitative and large-scale quantitative designs should be used in research on QoL in PP-SUPs, particularly among those with a parenting role.

## Background

Substance use problems (SUPs) affect the health and well-being of not only the person with the problem, but also their partners and families [1–3]. Substance use problems relate to consequences of substance use, such as physical and/or mental injuries, social and/or interpersonal problems, neglected major roles, and/or legal problems [4], and include a range of substances such as alcohol, opioids, cannabis, amphetamine/meth-amphetamine, and addictive drugs/medicines [5, 6]. Being the partner of a person with SUP involves being influenced by the consequences mentioned above. This study focuses on partners’ perspective on the SUP of the person with the problem, and hence the term substance use problem (SUP) is chosen over the diagnostic term ‘substance use disorder’ [6]. From the partners’ perspective there might exist a SUP when the use of substances disrupts the person’s tasks and functions that are to be taken care of in the family and / or interferes with the relationships between people [7]. Partners might experience the person with a SUP as having physical,- emotional,- and/or relational problems; problems at work or school; with the police because of the use of substances; or spending a lot of time using substances, or recovering from a hangover [8]. In Norway, Ireland and Australia, an estimated 10–30% of relatives, including partners, are affected by SUP in a close family member based on prevalence studies [5, 9, 10]. These problems may negatively affect various areas of relatives’ lives, such as poorer mental or somatic health [1, 8], social isolation, and poorer family conditions [11, 12]. Studies have also shown reduced lifespan (years of life) in close relatives of people with SUPs [1, 13]. In addition, studies report poorer socio-demographic conditions in close relatives: poverty, drop-out from school or work [1, 13, 14], and lower education levels have been reported in partners of persons with SUPs (PP-SUPs) compared to the general population [8].

The life areas reported above are essential dimensions of quality of life (QoL) in individuals, groups, and populations. Examining QoL can provide a broader perspective on individuals’ total situation than a more narrow focus on, for example, health or financial outcomes [15]. Understanding and assessing QoL in different populations may serve as a basis for the development of knowledge-based measures to promote health and prevent possible negative outcomes in different areas of QoL in vulnerable populations, such as PP-SUPs. Studies investigating QoL have increased in recent years. The concept, however, has been defined in various ways and not always clarified or defined when used in research; therefore, QoL measures can differ across contexts. Barcaccia et al. [16] analyzed the concept of QoL in their review and concluded that psychological, spiritual, and social dimensions should be included in addition to dimensions strictly related to physical health when evaluating QoL. These dimensions, understood as inherent in the QoL concept, are in line with previous definitions: physical, psychological, social, and relational dimensions [17], as well as environmental and existential dimensions [18, 19]. Environmental dimensions may be understood in line with Moons et al. [20], who described living condition domains, such as economy, housing, and security. Together, all these dimensions (i.e., physical, psychological, social, relational, spiritual/existential, and environmental) may constitute a more comprehensive understanding of the concept of QoL and is the perspective on QoL that informs this review.

There has been an increase in the number of studies examining QoL in which a key aspect is a subjective, self-reported assessment of QoL [16]. Many measurements have been developed to measure QoL. As each measure focuses on different dimensions [21], QoL measures are not homogenous. The health-related QoL measure Short Form 36 (SF-36) [22] measures physical and mental health and considered a generic measure across illness states [23]. Though the SF-36 measures the individual’s internal capability of life, other measures, such as the WHOQOL-BREF [18], measure inner life satisfaction or subjective enjoyment of life [24]. This indicates that health-related QoL measures tend to be more objective than subjective (as they ask questions such as whether the person has difficulty with mobility) than other measures such as rating of psychological well-being.

Relatives of persons with SUPs, including partners, have been recognized as an underserved population in healthcare [25], and QoL assessments can be useful in identifying those who struggle the most and need support or follow-up [15]. Examining the QoL of PP-SUPs will provide knowledge of their overall situation. Reviewing which QoL dimensions have been covered in studies of PP-SUPs’ quality of life will provide evidence on knowledge gaps that require further investigation. Synthesized knowledge on QoL in partners may serve as the basis for preventing negative outcomes, such as burdens and health risk, both for the partners and other relatives or family members (i.e., children) [8], as well as interventions to improve their well-being and QoL. Mapping (i.e. summarizing the range of evidence to describe breadth and depth) of the research field [26] regarding QoL of PP-SUPs will contribute to a broader picture of their situation. To the best of our knowledge, synthesis and summary of this evidence has not been conducted previously. Therefore, the overall aim of this scoping review was to examine the extent, range, and nature of the body of knowledge on QoL in PP-SUPs for the purpose of providing a synthesized summary of the evidence and to identify gaps in our knowledge of the QoL of PP-SUPs**.** The research questions are: 1. How has quality of life been investigated and measured with respect to PP-SUPs?, and, 2. How do PP-SUPs report their quality of life?

## Methods

A scoping review was conducted in collaboration with two experienced librarians (J.H. and E.S.) using systematic search methods. Scoping reviews have been used increasingly in health services research during the past few years [26, 27], as they are a suitable method in areas in which little research exists, or when existing studies appear heterogeneous in their results and conclusions. Systematic scoping reviews require formal methods but differ from other reviews in some ways. First, a scoping review aims to examine the extent, range and nature of the body of literature of a specific topic in a broader perspective and does not necessarily assess the quality of the included studies. Second, scoping reviews are suitable for identifying research gaps and may also provide a mechanism for summarizing and disseminating research findings to policymakers and health care providers. Identifying gaps may also lead to more research in a particular field [26, 28]. Due to a lack of a summary of knowledge on the QoL of PP-SUPs, such broad mapping is suitable for enabling an overview of the knowledge status in this area.

The choice of review method was also informed by initial searches in Google Scholar, followed by initial searches of the literature in two databases: EMBASE and PsycInfo. This search showed that studies investigating QoL in PP-SUPs were limited. To a large degree, studies were conducted among persons with SUPs, with a secondary aim to include their family members [29, 30]. The results reflecting QoL in these studies were also different and ambiguous and did not necessarily specify the rationale for using the same QoL instruments across the included subgroups of participants (i.e., patients and family member, herein also partners) [29, 30].

In order to map the broader literature, there was an agreement to include articles with multi-dimensional perspectives on QoL. There was also an agreement to extract associations with QoL that were statistically significant (i.e. 95% Confidence level.The approach for conducting systematic scoping reviews by Levac et al. [26] was used to guide the review based on the five-stage methodological framework developed by Arksey and O’Malley [28].

### Stage 1: Identifying the research question

The central questions guiding this scoping review were:

1. How has quality of life been investigated and measured with respect to PP-SUPs?
2. How do PP-SUPs report their quality of life?

### Stage 2: Identifying relevant studies

After the initial search in EMBASE and PsycInfo, six electronic databases were searched: EMBASE, Medline, PsycINFO, CINAHL, SocINDEX, and CENTRAL, with the last searches performed on June 23, 2017. No date limits were set. The search strategy included specifications of the *context* (substance use problems), *participants* (partners), and *concept* (quality of life) [31]. The search terms were then further identified. The *context* terms consisted of alcohol abuse, drug abuse, and drug dependence, with subgroups and different combinations. The *participants* terms consisted of partner, spouse, and significant other, also with subgroups and different combinations. The *concept* term consisted of quality of life, well-being, and life satisfaction.

Table 1 presents the search strategy that was used for EMBASE, which was adapted in minor ways for the other databases.

**Table 1:** Search strategy

Substance use problems (context):	
1 exp. Alcohol abuse/ (30694)	
2 exp. Drug abuse/ (98077)	
3 Substance abuse/ (48562)	
4 Alcoholism/ (112452)	
5 exp. Drug dependence/ (205497)	
6 ((drug* or substance* or alcohol*) adj2 (misus* or abuse* or addict* or depend* or overuse or problem* or “use disorder*”)).tw. (158120)	
7 ((opioid* or opiate* or opium or narcotic* or polydrug? Or heroin) adj2 (misus* or abuse* or addict* or depend* or overuse or problem*)).tw. (20773)	
8 (alcoholi* or “excessive alcohol use” or “drinking problem?” or “heavy drinking” or “binge drinking”).mp. (180840)	
9 ((beer or wine or liquor or spirits) adj (misus* or abuse* or addict* or depend*)).tw. (36)	
10 or/1–9 (418874)	
Partners (participants):	
11 exp. Spouse/ (13557)	
12 spous*.tw. (20290)	
13 exp. Marriage/ (57950)	
14 (marriage or “marital relations”).tw. (16392)	
15 (couple or couples*).tw. (68126)	
16 cohabit*.tw. (4618)	
17 “next of kin”.tw. (1650)	
18 (partner* or “other parent”).tw. (175877)	
19 (wife* or wives* or husband* or widow*).tw. (28711)	
20 “loved one*”.tw. (3646)	
21 ((significant or concerned) adj other*).tw. (4377)	
22 exp. Caregivers/ (58055)	
23 (caregiver* or care-giver* or “care giver*” or carer*).tw. (79780)	
24 (codependen* or co-dependen*).tw. (1120)	
25 Family/ (88179)	
26 famil*.ti. (230468)	
27 exp. Parent/ (208855)	
28 (parent* or mother* or father* or paternal or maternal).tw. (821969)	
29 or/11–28 (1431510)	
Well-being (concept):	
30 exp. “Quality of Life”/ (384374)	
31 (quality adj2 life).tw. (324553)	
32 (wellbeing or well-being or “well being”).tw. (82479)	
33 exp. Life satisfaction/ (7834)	
34 (satisfact* adj2 life).tw. (9640)	
35 (SEQOL or QOL or HRQL or WHOQOL* or EUROQOL*).tw. (62092)	
36 30 or 31 or 32 or 33 or 34 or 35 (519733)	
Combined search:	
37 10 and 29 and 36 (1468)	

### Stage 3: Study selection

#### Inclusion criteria

Quantitative and qualitative peer-reviewed, original, full-length research papers were included. Research papers identified through a manual search of key references and references known by co-authors were also included. The overall aim included summarizing knowledge status; thus, study protocols and conference papers in which the results had not been published in peer-reviewed journals were excluded. Because of limited time and resources, articles presented in languages other than English were also excluded. In addition, intervention studies and empirical papers which were not peer-reviewed were excluded.

#### Participants

The participants were present partners to persons with SUPs. The population may have been examined exclusively in “pure” partner studies, or as a subsample in a total sample of close relatives.

#### Concept

The key concept that was reviewed was the self-reported quality of life, including multidimensional dimensions, where at least physical and psychological health domains and social/relational domains occur.. Studies that had a very narrow focus on well-being, e.g., psychological distress only, were excluded.

#### Context

The context of the participants in the various studies was being a present PP-SUP. The substance use was characterized or described as problematic, heavy, or severe, or in terms of a medical diagnosis, and as the main condition. The context may or may not include a treatment situation.

#### Search strategy

**Fig. 1:**
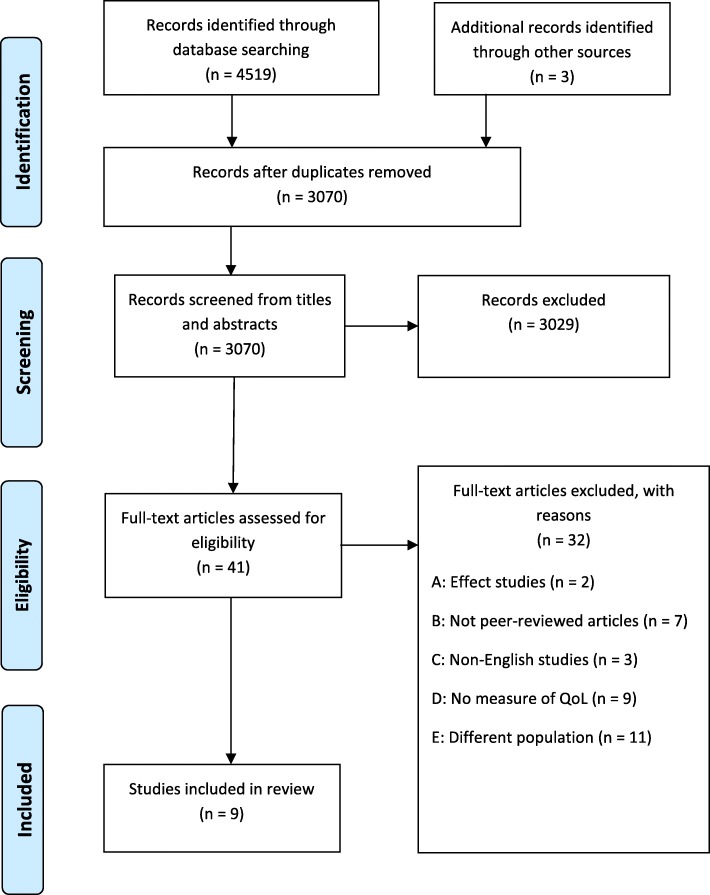
PRISMA flowchart

When performing the search strategy in the six different databases, a total of 4419 records were identified. These records were exported into EndNoteX8. Four records were identified through other sources, such as manually searching key references and feedback from co-authors. Duplicate records were then removed, resulting in 3070 records for screening the title and abstract. The screening was performed by two authors (BW and BB), who independently compared the titles and abstracts of each record with the inclusion criteria. BW and BB finally agreed to include 41 records as relevant studies for full-text screening. The records considered eligible for full-text screening were then distributed among two other authors, AS and MH, in addition to BB and BW, who independently screened the full-text studies to assess eligibility for inclusion in the review. One author (BB) screened the reference lists of the included studies. Of the 41 articles considered for inclusion, there was agreement on 36 (88%). In cases in which there was disagreement or doubt (12%), discussion meetings were held until an agreement was reached. In some cases, one of the other authors was consulted. Of the 41 screened full-text articles, 32 were excluded with reasons. The majority of these articles were excluded because the participants did not represent the relevant group (i.e. the persons with SUP had other main illnesses or conditions), they did not specifically present the results for the PP-SUP, the focus on well-being did not match our criteria for QoL, or well-being was measured in a very narrow way with, for example, only one QoL domain included. (e.g. stress, or well-being measured by using a depression scale only). This was also the case for the excluded qualitative articles, which in most cases focused on coping strategies as a measure of well-being. These articles were considered to diverge too much from the QoL domains. A total of nine articles were finally included in the review (Fig. 1).

### Stage 4: Charting the data

Quality of life issues related to PP-SUPs were analyzed by three authors (BB, BW, and KF) using steps from qualitative content analysis [32], including three main phases: preparation, organizing, and reporting. For this task, a structured data tool was used. One author (BB) extracted additional study characteristics, which were also reviewed by BW and then included in agreement between BB and BW. As this was a scoping review, study quality (e.g., risks of bias, study strength) was not considered [28]. The tables show the systematization and categorization of relevant topics from the results of the studies included in this review, reflecting the review questions.

### Stage 5: Collating, summarizing, and reporting the results

The main characteristics of the nine included studies are presented in Table 2. The studies were grouped by year of publication. Studies published in the same year were grouped in alphabetical order of first authors’ surnames.

**Table 2:** Studies examining QoL in partners to persons with substance use problems

Reference, year, and country	Aim and methods	Sample size and population	Type of substance use in the person with SUP	Test method for associations between having a partner with SUP and own QoL	Controlled for own SUP in PP-SUP in estimations of associations with QoL	QoL perspectives from results section
1. Brown et al. [36] 1995 Canada	To explore gender differences in married substance abusers admitted to treatment, particularly with a view to clarifying the relationship between client functioning and that of the spouse. Cross-sectional study QLQ (Quality of life Questionnaire)	*N* = 85 Gender of partners: 67 female and 18 male Mean age of partners: 37 years	Substance abuse (alcohol and other drug use)	Not performed	Not controlled for	Gender differences in partners’ QoL, with men scoring significantly lower on: • physical well-being • parent-child relations (less involvement with children and poorer parenting) • altruistic behavior (inability to provide support to others)
2. Barber et al. [35] 1997 Australia	To identify whether some coping responses are more likely than others to be associated with psychological adjustment in the partners of drinkers A multiple regression study Wolcott & Glazer’s 12-item Well-being scale	*N* = 60 Gender of partners: 57 women, 3 men Mean age of partners: 45 years	Heavy drinking	Standard multiple regression	Not controlled for	• No association between the use of negative behaviors towards the drinker and psychological well-being, irrespective of being used when the partner is drunk or sober • Negative behaviors towards the drinker when sober were marginally negatively related to psychological well-being • Positive behaviors towards the drinker had a marginally significant correlation with well-being
3. Dawson et al. [8] 2007 USA	To examine the association between partner alcohol problems and select physical and mental health outcomes among married or cohabiting women, before and after adjusting for potential confounders, and to compare these associations with those reflecting the impact of the women’s own alcohol-use disorders A cross-sectional, retrospective survey of a nationally representative sample of U.S. adults 18 years of age and older. Short Form-12 Health Survey Questionnaire, Version 2 (SF-12v2)-based physical quality of life. SF-12v2-based mental/ psychological quality of life	11,683 married or cohabiting women (PP-SUPs) Mean age of partners: 42 years	Alcohol problems	- Unadjusted (bivariate) regression models constructed to estimate the magnitude and significance of the associations between partner alcohol problems and the health outcomes. - Linear regression models for associations between numbers of stressors and QoL-scores.	Controlled for	• Significantly lower psychological QoL scores in women whose partners had alcohol problems • No significant difference in physical QoL between women with and without partner alcohol problems • Lower psychological QoL was significantly associated with higher level of own alcohol use of the participants • Partner alcohol problems were significantly associated with higher probability of being in fair or poor health and a lower mean psychological QoL scores
4. Casswell et al. [37] 2011 New Zealand	A first step in investigating relationships between exposure to heavy drinkers in respondents’ lives with measures of health status and well-being A cross-sectional general population survey European Quality of Life-5 Dimensions (EQ-5D) Personal Well-being Index (PWI)	*N* = 3068 (total sample) 29% had a heavy drinker in their lives Partners to heavy drinkers: 15% Mean age of partners: Not reported Gender of partners: Not reported (but for total sample 1232 males, 1836 females).	Heavy drinking	Proportional odds model used to predict relationship	Controlled for, but not reported on PP-SUPs exclusively	• Three-quarters of respondents who had a heavy drinking partner were in the highest exposure group (i.e., they were exposed to three or more heavy drinkers) • QoL in PP-SUP not reported • Women reported higher QoL than men • Poor QoL in respondents associated significantly with level of exposure to heavy drinker • The QoL domains activity, pain, and discomfort were significantly associated with high level of exposure to heavy drinkers • Older age, low income, low education level, higher levels of own drinking were significantly associated with lower QoL
5. Hussaarts et al. [30] 2012 Netherlands	Examine problem areas that patients with substance use disorders and their family members experience in terms of quality of relations, psychological problems, physical distress, and quality of life. Cross-sectional study European Quality of Life-5 Dimensions (EQ-5D)	*n* = 32 Dyads (persons with substance use disorders s and a family member)) were recruited from a substance abuse treatment program 22 partners Gender of partners: 23% males Mean age of partners: 45 years	Substance use disorder	Not performed	Not controlled for	• No QoL differences between subgroups (patients, partners, or parents) • Poor QoL in family members and in line with heroin addicts
6. Stenton et al. [34] 2014 Australia	Examine challenges to the health and well-being of families of people with alcohol problems A cross-sectional survey incorporating open-ended questions for qualitative analysis and closed-ended questions for quantitative analysis Quantitative part: Quality of life: single question, “How would you rate your quality of life?” Qualitative part: Open-ended questions about the seriousness of impact of their close relative’s drinking or substance misuse on their health and well-being	39 Al-Anon members 12 partners Gender of partners: Not reported Mean age of partners: Not reported	Problem drinking	Pearson’s product moment for correlation	Not controlled for	Quantitative part: • Higher levels of psychological distress in participants was associated with significantly poorer overall QoL • The participants’ satisfaction with a support group was associated with better overall QoL • QoL in PP-SUP not reported Qualitative part: • Poor relationships and lack of trust • Fear of aggression • Anxiety, sadness, and grief • Financial difficulties/poverty • Poor communication
7. Cicek et al. [29] 2015 Croatia	Comparing the quality of life (QoL) and family burden in relatives of patients with heroin dependence to healthy controls. A prospective case-control study World Health Organization Quality of Life Assessment-Brief (WHOQOL-BREF)	A total of 50 heroin-dependent patients and 50 of their relatives, and 50 healthy subjects and 50 of their relatives were included in the study Partners: 16% Gender of partners: Not reported, but 50% of total sample relatives were women Mean age of partners: Not reported (but 41 years for total sample of relatives)	Opioid dependence	Pearson product- moment correlation and Spearman’s rank correlation	Not controlled for	• No specific partner reports on QoL • QoL significant lower in family members of patients with heroin dependence than controls • All QoL subscale scores negatively correlated with the duration of illness
8. Jiang et al. [33] 2015 Australia	To identify which factors correlate with whether the respondent takes on this caring role for the person in their life whose drinking has most adversely affected them in the current year and to examine how caring for that person impacts the respondent’s quality of life and well-being, and use of services Cross-sectional survey European Quality of Life-5 Dimensions (EQ-5D) Personal Well-being Index (PWI)	778 respondents (total survey sample 2649) reported they were harmed because of the drinking of someone they knew (most harmful drinker; MHD). 67 partners Gender of partners: Not reported, but 67% of respondents harmed by MHD were women Mean age of partners: Not reported	Harmful drinking	Not performed	Not controlled for	• No QoL differences (EQ-5D) between subgroups (partners and others) • No significant differences in QoL (EQ-5D) in respondents who care for their ‘most harmful drinker’ (MHD) and those who did not • No significant differences in QoL between specific categories of MHD relationships • Personal well-being (PWI) significantly worse for people harmed by MHD than people who had no MHD in life • Poorer personal well-being (PWI) for caregivers of MHD in the household than non-caregivers
9. Nogueira et al. [38] 2015 Spain	To provide new empirical evidence about the effects of alcohol dependence on the health-related QoL of the dependent person and those around them using the general population as the control group Cross-sectional study Short-Form Health Survey-36 (SF-6D)	150 patients with alcohol dependence, 64 family members of patients with alcohol dependence, and 600 persons from the general population 67.7% partners Gender of partners: Not reported Mean age of partners: Not reported	Alcohol dependence	Logistic regressions	Not controlled for	QoL not reported for partners • Significantly lower QoL in family members than in general population (no specific partner reports on QoL) • Reduction in mean utility scores in SF-6D dimensions in family members, particularly in mental health and vitality, with a positive impact from physical function compared to the general population • Age and gender (being a woman) negatively correlated with QoL

### Findings

The findings of the review are presented according to the review questions. Table 2 reports the general information and major findings of the reviewed publications.

### How quality of life of PP-SUPs has been investigated and measured

Three of the nine included studies were conducted in Australia [33–35]. The remaining studies originated from Canada [36], New Zealand [37], the Netherlands [30], USA [8], Croatia [29], and Spain [38]. The studies were spread over a wide timeframe, with two studies published before 2000 [35, 36] and seven published after 2010. The majority of the studies aimed to examine the impact of SUPs on close family members, with QoL as one of the primary outcomes. In four of the studies, the participants were recruited when their partner was in treatment [29, 30, 36, 38], in one study the participants were recruited through newspaper advertisements [35], and in one study the participants were recruited in mutual aid support groups (Al-Anon Family Groups and FDH, a program of the Self-Help Addiction Resource Centre (SHARC), both in Australia) [34].

#### Methods

Eight of the studies had a quantitative design. Three studies were larger, general population surveys [8, 33, 37]. One was a case-control study [29], and four had a cross-sectional design [30, 35, 36, 38]). One of the studies had a mixed methods design in which QoL was included in the qualitative part [34]. No purely qualitative studies were found that met the inclusion criteria.

#### QoL measures

The instruments used, QoL domains, and studies are listed in Table 3. A wide range of instruments were used. Five of the studies used instruments covering health-related QoL. Two of these five studies used two different versions of the SF-36: the SF-12 [8] and SF-6D [38]. The SF versions; − 36/− 12/−6D are described both as generic health measures and health related quality of life measures. These instruments cover eight and six domains on mental and physical health, respectively [39–42]. As for the three studies using EQ-5D [30, 33, 37], a QoL measure covering five dimensions of health [43], two of them [33, 37] supplied the Personal Well-being Index PWI [44, 45] to capture measures of life and life satisfaction as a whole. Another study [36] used both the PWI and the Quality of Life Questionnaire (QLQ), which includes eight scales on well-being [46].

**Table 3:** QoL measures and domains

Instrument	QoL domains	Study
SF-12 (12 items) [39]	Physical functioning Role physical Bodily pain General health Mental health Role emotional Social functioning Vitality	[8]
SF-6D (6 items) [42]	Physical functioning Role limitations Pain Mental health Social functioning Vitality	[38]
EQ-5D (5 items and a VAS on current overall health) [43]	Mobility Self-care Usual activities Pain/discomfort Anxiety/depression	[30, 33, 37]
Personal Well-being Index (PWI) (8 items and a single question of satisfaction with life as a whole) [45]	Standard of living Personal health Achieving in life Personal relationships Personal safety Community connectedness Future security Spirituality/religion	[33, 37]
WHOQOL-BREF (27 items) [18]	Physical Psychological Social relationships Environment	[29]
Quality of Life Questionnaire (QLQ) (192 items) [46]	Material well-being Physical well-being Personal growth Marital relations Parent/child relations Extended family relations Extramarital relations Altruistic behavior	[36]
Wolcott & Glezer’s well-being scale (12 items) [48]	Standard of living Relationship Personal feelings of self-worth	[35]
A single question on overall QoL: *How satisfied are you with your life?*	Open-ended questions about the seriousness of the impact of their close relative’s drinking or substance misuse on their health and well-being	[34]

The rest of the quantitative studies (*n* = 3) measured QoL using different instruments covering a range of QoL with at least health, social, and relational domains. One study [29] used a Turkish version of the WHOQOL-BREF, which includes eight domains on physical and psychological health and social relations [47]. Some of the instruments covered existential, environmental, and living standard domains. Barber and Gilbertson [35] used Wolcott & Glazer’s 12-item well-being scale [48] covering standard of living domains, relational domains, and feelings of self, and including some questions on health. Finally, the study using a single question asking the participants to rate their perceived overall QoL [34] did not report which domains this was meant to cover. They did, however, include a qualitative part with questions about health and well-being, which was supposed to cover QoL.

#### Population

Only three of the nine included studies [8, 35, 36] were conducted among PP-SUPs exclusively. The six remaining studies [29, 30, 33, 34, 37, 38] were conducted among other close relatives to persons with SUPs, including a percentage of PP-SUPs.

Only four of the nine selected studies provided socio-demographic details of the PP-SUPs regarding age and gender, and three of these five studies also reported on minor children living in the household. In the three studies evaluating PP-SUPs exclusively, the mean age was 42 years [8], 45 years [35], and 37 years [36]. Hussaarts et al. [30] reported a mean age of 45 years in PP-SUPs in the total sample of relatives. The same studies reported the gender of PP-SUPs. There was a large proportion of female partners (average 88%). One study had 77% female partners [30]; in the rest of the studies, the proportion of women ranged from 79% [36] to 95% [35]. Finally, one study [8] was conducted among females only. No studies conducted exclusively among men were found. Five of the remaining studies did not report on socio-demographic variables, such as age and gender, in PP-SUPs specifically [29, 33, 34, 37, 38]. In addition, when reporting on demographic variables, three of the nine included studies reported that some of the PP-SUPs were parents to minor children living in the household, namely 58% [36], 50% [8], and 54% [30].

### How PP-SUPs report their QoL

Three of the nine studies reported QoL exclusively in PP-SUPs [8, 35, 36], but varying QoL results were reported. Only one of the included studies controlled for own SUP in PP-SUPs in estimations of associations with QoL. In this study, a survey investigating the impact of partner alcohol problems in American women [8], lower QoL was found in PP-SUPs than the general population. Lower psychological QoL was significantly associated with higher level of own alcohol use of the participants. The partners’ alcohol use appeared however to have at least as great negative effect on QoL in PP-SUPs as the participants’ own alcohol use. In another study exploring gender differences in spouses of partners in treatment [36], male partners reported lower QoL than female partners. In addition, the male partners’ lower QoL was associated with poor relationships with their children and poor social support skills. The third study examining partners living with a heavy drinker [35] found no associations between PP-SUPs’ psychological well-being and negative behavior towards the drinker, regardless of whether such behavior was present when the partner was drunk or sober. The authors underlined that well-being may also be determined by other factors.

The two studies reporting PP-SUPs as part of the overall study [30, 33] also reported on partners’ QoL specifically. When examining differences between subgroups, they both found that the PP-SUPs’ QoL did not differ significantly from the other subgroups of participants, such as SUP patients or other relatives [30, 33] . These studies also presented various results regarding QoL. Both studies [30, 33] found that the relatives [30, 33], including PP-SUPs, reported significantly lower QoL than the general population. The authors [30, 33] proposed that the poor QoL may have been due to the strains and burdens of living with or caring for a person with a SUP. Further, when examining factors associated with QoL, both studies [30, 33] found that poor QoL in relatives [30, 33] is associated with the severity of the substance use in the person with SUPs. Jiang et al. [33] also found that caring for the person with alcohol use is negatively associated with QoL.

The results of the last four studies [29, 34, 37, 38] did not differ between subgroups, but reported on relatives of people with SUP as a whole, though they reported a percentage of PP-SUPs in their results. All of these studies found poorer QoL in relatives of people with SUP than in the general population or controls, with various factors that may explain this difference. In a population survey examining the negative impact of exposure to others’ drinking, Casswell et al. [37] found a reduction of QoL that was significantly related to an increase in the level of such exposure, and that 75% of the participants represented in the group reporting highest exposure were PP-SUPs. They also found a strong association between higher QoL and being a woman, though being unemployed/sick and on low income was associated with lower levels of QoL for all participants. In the estimations of associations with QoL in the participants, one of the studies found that higher levels of the relatives’ (including PP-SUPs) own drinking were significantly associated with lower QoL [37]. The other study [34] found no significant associations between the relatives’ (including PP-SUPs) own substance use and measures of QoL. In a study of 150 alcohol-dependent persons and 64 family members of alcoholics, Nogueira et al. [38] found that poor QoL in family members was generally associated with higher age and being a woman, whereas education and living with a partner positively correlated with QoL.

The remaining studies including relatives [29, 34] in general reported a negative correlation between low QoL in relatives and duration of heroin dependence, age, and education of both patients and relatives, and the onset age of heroin use. Stenton et al. [34] found that poor QoL in relatives was associated with psychological distress, whereas better QoL was associated with the level of satisfaction with attendance in a mutual aid support group. In this study they found no significant associations between relatives’ (own) alcohol consumption and measures of QoL or well-being.

## Discussion

The studies included in this scoping review originated from a wide range of countries, and the majority were conducted after 2010, which indicates an increased interest in research focusing on both QoL and PP-SUPs. One study with a mixed methods design [34] was included and no qualitative studies matched our inclusion criteria of exploring PP-SUP experiences with QoL were found in the research emerging after 2010. The majority of the studies used established and generic instruments when examining QoL. However, these instruments are based on different concepts and theoretical perspectives of QoL; therefore, findings cannot be consistently compared across studies. Many studies also utilised different comparison groups (e.g. the general population, people who had no person with SUP in their life, controls, patients vs. partners, vs. parents, etc.), which shows a heterogeneity between studies.

As for the instruments used to measure QoL, two of the studies that used the EQ-5D, a QoL instrument solely covering health domains, added the PWI with broader domains [33, 37]. Three other studies using EQ-5D [30], SF-12 [8], and SF-6 [38], did not include other instruments to add additional dimensions other than health when reporting on QoL. As health-related QoL measures often refer to an illness and treatment of patients [23], and tend to be more “objective” as they target specific functioning levels, they may have been considered suitable when examining health-related QoL in persons with SUPs. The question remains whether this reflects the QoL dimensions that are most important to family members or relatives in general or PP-SUPs specifically; these persons may experience a difficult life situation but are not necessarily ill. The remaining four quantitative studies [11, 29, 35, 36] used instruments covering a wider range of QoL domains in addition to health. This included at least social and/or relational domains, and some of them even existential or environmental domains. Conclusively, though all the instruments covered the health domain, only half of the studies made use of instruments that embrace QoL in a broader manner, including at least social and relational dimensions. Therefore, the findings are heterogeneous because researchers are not consistently using the same measures. Many studies only include particular dimensions of QoL rather than a more comprehensive concept of QoL. The mixed methods study [34], which included an overall question about quality of life, introduced a broader perspective on QoL by including a single qualitative question about the participant’s well-being in different areas. This qualitative information can provide further contextual information and explanations for quantitative findings and may be useful to include in future research on QoL.

For future research of PP-SUPs, QoL measures that capture the broader dimensions of QoL are recommended. In addition, generic instruments that provide the possibility of cross-population comparisons would be useful. It also seems that multi-dimensional QoL forms could better capture variations in the life situation of these partners and provide a more holistic understanding of their overall life situation’s impact on their QoL. Of the instruments included in the studies in this review, only WHOQOL-BREF include social, relational, and existential dimensions in addition to physical and mental health. To capture more dimensions than those covered by the highly health-specific instruments (e.g., EQ-5D, SF-6D, and SF-12), they can be used together with PWI, which also includes social, relational, and existential dimensions.

Only three of the included studies focused on PP-SUPs exclusively. However, two of the other studies including PP-SUPs as a subgroup did report on their QoL. The remaining studies did not differ between subgroups when presenting QoL results, but presented the QoL results to apply to the entire sample. Thus, more research is needed that focuses on PP-SUPs exclusively. In the case of socio-demographic variables, the average age of PP-SUPs is relatively low (42 years), which may reflect the fact that in three of the nine reviewed studies over half of the participants were described as caring for minor children. Parenting was however not themed specifically, which indicates a knowledge gap. Women comprised more than 3/4 of the participants on average. This is in line with other research conducted among PP-SUPs in which the proportion of women has often been higher [13, 14, 49]. Though the findings by Dawson et al. [8] represent female partners only, and the rest of the studies reported a majority of female PP-SUPs, no studies were found that focus exclusively on male PP-SUPs. Therefore, there is a gap in knowledge on the QoL of male partners, especially as male partners have reported very poor QoL [36], and further research on male partners’ QoL is needed.

A key finding was that, in the majority of the studies, substance use by the person with SUPs was the factor that related most to poor QoL among the participants, including PP-SUPs [8, 30, 33, 35]. An association was found between severity of SUPs and poorer QoL in PP-SUPs [30, 33, 37]. The majority of studies also reported that the participants, including PP-SUPs, described a lower QoL than the general population. These findings indicate that substance use itself has a great impact on the PP-SUPs’ QoL. One study found that PP-SUPs’ QoL was more affected by SUPs in a partner than the PP-SUPs’ own substance use [8]. In addition, several of the studies showed that PP-SUPs had equally poor QoL as people with SUPs. This indicates a very stressful life situation. Although they are not by definition ill, long-term and serious substance use problems have a major impact on PP-SUPs’ QoL. Using a broad measure of QoL that includes at least health, social, and relational dimensions, rather than pure health-specific QoL measures, in future research could be more suitable for capturing partners’ life situations.

The results describing associations with QoL varied greatly. In addition to the impact of SUPs on QoL, there are some specific findings that need to be discussed and addressed with respect to PP-SUPs. Firstly, only one of the studies controlled for PP-SUPs’ own substance use in their estimations of associations between having a partner with SUP and lower QoL. This study found a significant association between lower QoL in PP-SUPs and own substance use. Two other studies, conducted among relatives such as people exposed to heavy drinkers [37] and family members of people with alcohol problems [34], respectively, also examined associations between the relatives’ own substance use and QoL, but did not report on PP-SUPs exclusively. One of these studies [37] found that substance use in the participants was associated with QoL. Hence, we cannot know how the PP-SUP’s own substance use, and their partners’ substance use, respectively, impact on partners’ QoL. It is interesting however to see that male PP-SUPs have lower QOL (than female PP-SUPs) in a study that does not control for own consumption [36], whereas female PP-SUPs have lower QOL (than the general population) in the study that does control for own consumption [8]. This indicates that own consumption may be an important confounder to control for. Controlling for own substance use may be particularly important for disentangling gender differences in associations between being a PP-SUP and QoL. This result represents a gap which needs to be further examined in future research. Secondly, the fact that three of the studies [8, 30, 36] reported on whether the partners had minor children living in the household must be considered when presenting the associations with QoL. This is especially important when poor QoL was found to be associated with being male and poor parent/child relations [36]. Several studies have shown that the parent/child relationship is disrupted due to SUPs in a parent [2, 12, 50]. The PP-SUPs’ poor QoL may influence the capacity to fill the parenting role. Taken together, these findings underline the importance of paying attention to PP-SUPs that also have a parenting role. Possible negative outcomes for partners is relevant not only to tailor support for their own sake, but also to enhance parenting and prevent negative outcomes for the children. Conversely, better parenting ability may mutually reinforce the parent’s overall situation [51].

As for PP-SUPs’ positive associations with QoL, one study conducted in 1997 found that positive behavior towards their partner with SUPs was associated to some degree with PP-SUPs’ QoL [35]. Though these are also important findings to address clinically, a minority of studies seem to have examined other factors associated with QoL in PP-SUPs, both positive and negative. Studies reporting on PP-SUPs’ positive associations with QoL seem limited, and there is a gap in knowledge in this area. Studies investigating and exploring QoL qualitatively and quantitatively in PP-SUPs are also needed.

## Conclusions

This scoping review shows that poor QoL of PP-SUPs is associated with the partner’s SUP, and this should be addressed by health personnel who need to increase focus on PP-SUPs’ QoL when patients with such problems are in treatment. This result is also relevant for policymakers. PP-SUPs should be included in the development of national guidelines based on larger scale research. The importance of national guidelines being evidence-based is emphasized; therefore, such evidence must be valid and reliable. Currently, the evidence is ambiguous, and there is a need for larger generalizable studies. Furthermore, there is a need for more research among PP-SUPs who also are parents to minor children as poor QoL may affect parenting. Gender issues should also be taken into consideration when conducting such studies.

This review has revealed some important gaps with respect to knowledge about QoL in PP-SUPs. First, due to the minority of studies conducted among PP-SUPs exclusively, there is a need for further research examining QoL in this group. QoL has been studied in PP-SUPs to a limited extent. In addition, a variety of QoL instruments with various dimensions of the concept included have been used in studies of this particular population. This indicates a challenge in making comparisons between groups. On the one hand, generic instruments that may compare this population with the general population or other at-risk-groups exist, whereas on the other hand, generic instruments may fail to capture specific areas of importance to certain populations, such as PP-SUPs. Thus, in addition to studies with larger scaled quantitative designs, a need exists for research exploring QoL qualitatively in this particular group, especially among PP-SUPs who also have a parenting role.

### Strengths and limitations

The main strength of the present scoping review is the comprehensive database search without a date limit. The search was conducted with comprehensive search terms, which identified a large number of studies. This strength is largely due to close collaboration with two highly experienced academic librarians from different disciplines during the search. The titles and abstracts were screened thoroughly and systematically performed by two authors. Full-text studies were further screened by four authors, followed by reference lists and discussion meetings, which also assured against a loss of relevant studies**.** However, studies in languages other than English were not included, which may have caused some relevant records to be missed. Though the search was performed in six databases, this number is not exhaustive. However, the selected databases and the search performed were advised by experienced academic librarians in order to cast as wide a net as possible regarding the population, concept, and context.
